# WNT signaling in midbrain dopaminergic neuron development and cell replacement therapies for Parkinson’s disease

**DOI:** 10.1186/2193-1801-4-S1-L49

**Published:** 2015-06-12

**Authors:** Ernest Arenas, Carmen Saltó, Carlos Villaescusa

**Affiliations:** Karoliska Intitute, Sweden

**Keywords:** WNT, dopaminergic neurons, Parkinson’s disease

Parkinson’s disease (PD) is a neurodegenerative disease characterized by the loss of midbrain dopamine (mDA) neurons. Clinical trials using human embryonic midbrain tissue for transplantation have provided proof of concept that cell replacement therapy (CRT) can lead to not only symptomatic relief, but also changes in the course of disease and withdrawal of medication. Human pluripotent stem cells are currently regarded as the main candidate cell type for CRT because they are readily available, expandable, and can be standardized and differentiated into mDA neurons capable of inducing functional recovery in animal models of PD. However, protocols for mDA differentiation are still far from optimal and require further improvement. We previously found that members of the Wnt family of morphogens regulate multiple aspects of mDA neuron development [1]. Different branches of the Wnt signaling pathway, such as Wnt/β-catenin, activated by Wnt1, and Wnt/PCP, activated by Wnt5a, have been thought to regulate separate or opposing functions. However, we found that Wnt5a cooperates with Wnt1 to promote mDA neurogenesis and that Wnt1 cooperates with Wnt5a to promote the differentiation of postmitotic mDA neuroblasts [2]. We are currently applying this knowledge to improve protocols for the differentiation of human stem cells into mDA neurons suitable for transplantation and functional recovery in animal models for PD [3].
